# Ginsenosides, salidroside, and syringin complex exhibits anti-fatigue in exhaustive exercise rats

**DOI:** 10.7150/ijms.99889

**Published:** 2025-01-01

**Authors:** Yung-Chun Wu, Yu Zhi Lian, Hongwei Zhao, Lei Wang, Deshan Ning, Jane C.-J. Chao

**Affiliations:** 1 School of Nutrition and Health Sciences, Taipei Medical University, Taipei 110, Taiwan.; 2 Infinitus (China) Company Ltd., Guangzhou 510405, China.; 3 Master Program in Global Health and Health Security, Taipei Medical University, Taipei 110, Taiwan.; 4 TMU Research Center for Digestive Medicine, Taipei Medical University, Taipei 110, Taiwan.; 5 Nutrition Research Center, Taipei Medical University Hospital, Taipei 110, Taiwan.

**Keywords:** Chinese herb, exercise performance, lactate clearance, antioxidation, anti-inflammation, rats

## Abstract

Excessive exercise can lead to fatigue, consequently affect exercise performance, and further have an adverse impact to human health. The synergistic effects of ginsenosides, salidroside, and syringin on improving exercise performance remain unknown. Hence, the effects of Chinese herb powder (CHP) which consisted of bioactive compounds such as ginsenosides (Rg1, Re, and Rb1), salidroside, and syringin on exercise performance, energy metabolism, tissue damage, antioxidant activity, and inflammatory cytokine were investigated in exhaustive exercise rats. Male Sprague-Dawley rats aged of 8-week-old were randomly assigned into four groups: control (normal, N), low-dose (L, 310 mg/kg bw), medium-dose (M, 620 mg/kg bw), and high-dose (H, 1550 mg/kg bw) groups. The intervention groups were orally given CHP daily for successive 30 days. Abdominal arterial blood, liver, and gastrocnemius muscles were collected 4 hours after exhaustive exercise for further analysis. The high-dose CHP group increased the time to exhaustion, decreased serum lactate level, increased serum superoxide dismutase activity, and decreased liver interleukin-6 concentration. Therefore, CHP exhibits an anti-fatigue effect for prolonging the time to exhaustion through improving lactate clearance, and to a lesser extent, enhancing the capacity of antioxidation and anti-inflammation.

## Introduction

Fatigue is defined as a sense of tiredness, a lack of energy, and the feeling of exhaustion, which could be related to the difficulty in performing voluntary tasks, and could occur at rest, after the beginning of exercise, and during or after exercise 1-3. Furthermore, increased work pressure and strenuous exercise can lead to fatigue 2-4, fatigue can result in a lack of concentration, slower reaction time, and reduced muscle strength, and eventually affected athletic endurance performance 5. Therefore, prolonging the time to fatigue and facilitating post-exercise recovery are crucial.

There are three main theories including the exhaustion theory, clogging theory, and radical theory to explain the mechanisms of fatigue induced by exercise 6. The exhaustion theory explained that energy storage molecules such as ATP, phosphocreatine, and glycogen are extensively consumed during endurance exercise, and led to a decline in skeletal muscles 7. The clogging theory suggested that the accumulation of serum lactate and blood urea nitrogen (BUN) led to fatigue during intense exercise 8, 9. The radical theory proposed that high-intensity exercise generated excessive free radicals to cause metabolic imbalance and fatigue 7. Therefore, to avoid fatigue in a short period could be achieved by increased energy production from the utilization of carbohydrate and fatty acids, raised glycogen storage, improved clearance of energy metabolites, and elevated activity of antioxidant enzymes such as superoxide dismutase (SOD), catalase, and glutathione peroxidase for reducing oxidative damage caused by exercise 10, 11.

Ginsenosides Rb1, Re, and Rg1, the most studied substances, are active compounds in American ginseng, and showed anti-inflammation, antioxidation, and anti-fatigue actions 12-15. Intragastric intervention of ginsenoside Rb1 (50-100 mg/kg bw) for 4 weeks prolonged the swimming time to exhaustion, decreased serum creatine kinase (CK) activity and reduced hepatic malondialdehyde (MDA) level in exhaustive exercise mice 16. Salidroside as an active compound was extracted from rhodiola, and has been well-known for its antioxidation and anti-inflammation 17, 18. After 15-day oral administration of salidroside (180 mg/kg bw), the exhaustive mice prolonged the longest swimming time, increased glycogen levels in liver and gastrocnemius muscles, but reduced serum lactate level 19. Syringin is an active phenolic compound of *Eleutherococcus senticosus* known as Siberian ginseng, and was found to exert antioxidation, anti-inflammation, and anti-fatigue 20. Syringin was identified as one of the most potentially effective anti-fatigue compounds in Siberian ginseng 21.

However, most previous studies investigated these active compounds individually against fatigue, and did not further explore whether the intervention with a mixture on anti-fatigue could have synergistic effects. The aim of this study was to assess whether ginsenosides, salidroside, and syringin powder mixture affected exercise performance through the improvement of energy metabolism, tissue damage, oxidative stress, and inflammation in exhaustive exercise rats.

## Materials and Methods

### Materials and reagents

Chinese herb powder (CHP, named E-Getic authentic herbal mix) was provided by Infinitus (China) Co. Ltd. (Guangzhou, China). CHP was extracted by aqueous extraction, the extract contained 0.124% ginsenosides containing Rb1 (Supplementary Figure S1A), Re (Supplementary Figure S1B), and Rg1 (Supplementary Figure S1C), 0.196% salidroside (Supplementary Figure S1D), and 0.054% syringin (Supplementary Figure S1E). The kits for glycogen (K646-100), lactate (K607-100), ammonia (K370-100), and free fatty acids (FFA, K612-100) were purchased from BioVision Inc. (Milpitas, CA, USA). Insulin concentration kit (10-1251-01) was bought from Mercodia AB (Uppsala, Sweden). The kits for MDA (10009055) and SOD activity (706002) were obtained from Cayman Chemical (Ann Arbor, MI, USA). The enzyme-linked immunosorbent assay (ELISA) kit for rat interleukin-6 (IL-6, DY506) was bought from R&D Systems, Inc. (Minneapolis, MN, USA).

### Animals and design

Thirty-two 7-week-old male Sprague-Dawley rats with an initial weight of 200-250 g were bought from BioLASCO Taiwan Co., Ltd. (Taipei, Taiwan). All animals were housed individually in the Laboratory Animal Center of Taipei Medical University (TMU), and maintained in a 12-h light/dark cycle with a relative humidity of 65 ± 5% at 22 ± 2℃. The rats were acclimated for one week, and randomly assigned into four groups (*n* = 8/group): normal (N), low-dose (310 mg/kg bw/d, L), medium-dose (620 mg/kg bw/d, M), and high-dose (1550 mg/kg bw/d, H) groups. All the intervention groups were administered orally by gavage feeding for 30 days continuously. CHP was dissolved in distilled water and prepared to the stock solution with the concentration of 600 mg/mL. The rats were fed with distilled water only (the N group) or designated CHP concentrations in distilled water (the CHP treated groups) by gavage, and the total volume of oral gavage was 1 mL for each rat.

During the experimental period, fasting serum was collected on days 0 and 23, forelimb grip strength was measured on days 0 and 26, lactate accumulation was performed on day 26, and exhaustive running was done on day 30. Before conducting lactate accumulation and exhaustive running, all the rats were acclimated to the treadmill for 3 times weekly by gradually increasing the velocity from 10 to 25 m/min for 10 min. All the animal protocols were approved by the Institutional Animal Care and Use Committee of TMU (approval no. LAC-2021-0031), and followed by the Guide for the Care and Use of Laboratory Animals (8th edition, National Academies Press, Washington, DC, USA).

### Forelimb grip strength

The forelimb grip strength is considered to be an indicator of athletic performance, and was widely used to evaluate the effect of anti-fatigue 22. The forelimb grip strength was determined by BIO-GS3 (Bioseb, Vitrolles, France), and the change of the forelimb grip strength was calculated after 4-week intervention of CHP. Furthermore, grip strength was calibrated by body weight to rule out its possible effect. Grip strength was measured 5 times, and the mean was calculated by 3 measures after excluding the maximum and minimum values.

### Lactate accumulation

The rats were orally given CHP by gavage 30-min before running on the treadmill, and started running with a velocity at 10 m/min for 10 min, 20 m/min for the following 10 min, and 25 m/min for the final 10 min. The rats rested for 30 min after running. Serum lactate level was evaluated colorimetrically, and measured at three time points on day 23 (L0), immediately after 30-min running on day 26 (L1), and 30-min rest after running on day 26 (L2). Blood samples (1 mL) was collected at 3 time points from the tail vein, and centrifuged at 1200 ×*g* for 10 min at 4℃. Serum was separated in the supernatant. Serum samples (2 μL) were diluted to 50 μL, and incubated with reaction mix at room temperature for 30 min. The absorbance was determined at 570 nm to calculate lactate concentration.

### Exhaustive running test

The rats were orally given CHP by gavage 30-min before running on the treadmill, started running at an initial velocity of 10 m/min, gradually increased 5 m/min every 10 min up to 30 m/min, and remained this speed until fully exhausted to achieve the maximal exercise capacity. If the rats remained running after an hour from the beginning, the treadmill slope was increased from 0 to 10 degrees, and the rats continuously ran on such slope until reaching exhaustion. The exhaustion of the rats was defined as 5 electric shocks without further getting up to run and failing to keep up with the speed of the treadmill. According to the previous study, the rats ran to exhaustion on the treadmill of 10-degree incline and at a speed of 30 m/min for reaching the relative intensity of 70%-75% VO_2_max 23. The time to exhaustion was recorded. All rats were anesthetized by intraperitoneal injection with Zoletil^®^ 50 (Tiletamine + Zolezepam, 50 mg/kg bw)/Rompun^®^ (10 mg/kg bw), and sacrificed 4 h after exhaustion. The anesthesia method was commonly used in the rodent study for the measurement of oxidative stress markers such as MDA and SOD 24, 25 or inflammatory marker such as IL-6 26 because of the effectiveness and minimal discomfort. Liver and gastrocnemius muscles were obtained for further analysis. Serum samples at three time points on day 23 (E0), immediately after exhaustive running on day 30 (E1), and 4-h after exhaustive running on day 30 (E2) were collected. To avoid the interference of blood withdraw with exercise, fasting blood samples (1 mL) from the tail vein at E0 and E1 were collected a week before and immediately after the measurement of the forelimb grip strength and time to exhaustion. Serum was separated as mentioned above. Blood samples (1 mL) were obtained from the abdominal aorta at E2, and centrifuged at 1000 ×*g* for 10 min. Serum was further stored at -80℃ for the measurement.

### Energy metabolism

Serum BUN, creatinine (CRE), and uric acid (UA), and glucose levels as energy metabolites for energy utilization parameters were determined using the AU5800 series clinical chemistry analyzer (Beckman Coulter Inc., Brea, CA, USA). Serum ammonia and FFA were evaluated colorimetrically according to the manufacturer's instructions. The absorbance was assessed at 570 nm to calculate serum ammonia and FFA.

Serum insulin concentration was determined using ELISA according to the manufacturer's instructions. Serum insulin concentration was calculated by the standard curve.

Glycogen was measured by an enzymatic colorimetric method in liver and right gastrocnemius muscles. Tissue samples (10 mg) were homogenized using TissueLyser II (Qiagen, Hilden, Germany), and centrifuged by 18,000 ×*g* for 10 min at 4℃ to collect the supernatant. The assay was conducted according to manufacturer's protocol. The absorbance was evaluated at 570 nm to calculate glycogen content.

### Tissue damage

Tissue damage was assessed by serum biochemical markers and tissue histopathological staining. Serum CK, lactate dehydrogenase (LDH), glutamic oxaloacetic transaminase (GOT), and glutamic pyruvic transaminase (GPT) activities were determined using the AU5800 series clinical chemistry analyzer (Beckman Coulter Inc.).

Liver and right gastrocnemius muscles were fixed in 10% formalin solution for 24 h, dissected, and embedded in paraffin after gradient dehydration. The 5-μm serial sections were stained with hematoxylin and eosin. The severity of liver and muscle damage was evaluated by an experienced pathologist according to the method of Shackelford 27. Scores were divided into 6 grades from 0 to 5 which indicate no lesions, minimal lesions (1%), mild lesions (1%-25%), moderate lesions (26%-50%), moderately severe lesions (51%-75%), and extremely severe lesions (76%-100%). A total score in liver was the sum of 6 pathological items (apoptosis of hepatocytes, the infiltration of mixed inflammatory cells, hepatocellular necrosis with the infiltration of inflammatory cells, the proliferation of hepatic oval cells, vacuolar degeneration of hepatocytes, and the infiltration of inflammatory cells with hepatic congestion). A total score in gastrocnemius muscles was the sum of 3 pathological items (the infiltration of mixed inflammatory cells, regenerating myofibers, and degenerated/necrotic myofibers).

### Oxidative stress markers

Lipid peroxide MDA level and antioxidant SOD activity in serum, liver, and gastrocnemius muscles were measured colorimetrically. Tissue samples (100 mg) were mixed with 1 mL phosphate-buffered saline, homogenized using TissueLyser II (Qiagen, Hilden, Germany), and centrifuged by 13,000 ×*g* for 10 min at 4℃. The supernatant was obtained for measurement. The absorbance was determined at 535 nm to evaluate MDA level.

Serum or tissue homogenate samples (10 μL) were added to radical detector (200 μL) in a 96-well microplate, incubated with xanthine oxidase (20 μL), and placed on a shaker for 30 min at room temperature. The absorbance was assessed at 440-460 nm to calculate SOD activity.

### Inflammatory marker

Pro-inflammatory marker IL-6 levels in liver and gastrocnemius muscles were assessed by ELISA. Tissue samples were prepared the same as the measurement of oxidative stress markers described above. The assay was performed followed by manufacturer's protocols, and the absorbance was evaluated at 450 nm to calculate IL-6 level. Protein content was quantitated by Lowry's method 28.

### Statistical analysis

Data are presented as mean ± standard error of the mean (SEM). Statistical comparisons were analyzed using SAS 9.4. (SAS Institute Inc., Cary, NC, USA). The differences among the four groups were compared by one-way repeated measures ANOVA and Duncan's new multiple range test. The *p*-value < 0.05 was concerned statistically significant.

## Results

### High-dose CHP supplement increased time to exhaustion

No significant differences were found in initial and final body weight, grip strength at weeks 0 and 4, and the changes of grip strength between week 4 and week 0 among the four groups (Table 1). The high-dose CHP group significantly increased the time to exhaustion compared to other groups (*p* < 0.05).

### CHP supplement enhanced serum lactate clearance

Serum lactate level was significantly increased after exercise (L1) compared to that before exercise (L0), and reduced after 30-min rest (L2) compared to that after exercise (L1) in all the groups (Table 2). The intervention groups at all the doses decreased serum lactate level after 30-min rest (L2) compared to the normal group (*p* < 0.05).

### Energy metabolism was altered after exhaustive exercise

**Figure 1:**
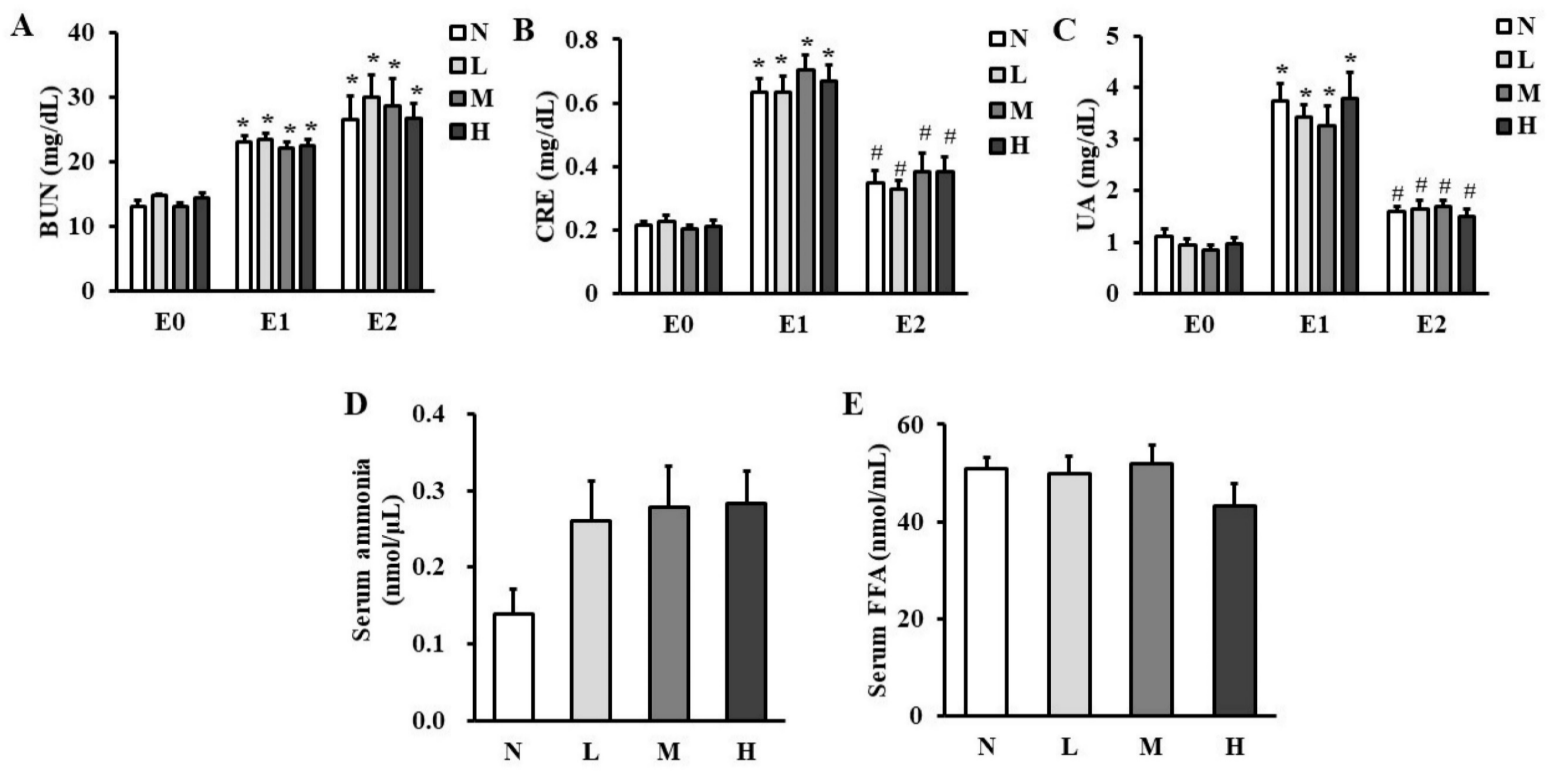
Effect of CHP on energy metabolic markers. (A) serum BUN, (B) serum CRE, (C) serum UA, (D) serum ammonia, and (E) serum FFA in exhaustive rats. Data are indicated as mean ± SEM (*n* = 8). ^*^p < 0.05 vs E0 and ^#^*p* < 0.05 vs E1. CHP: Chinese herb powder, N: normal group, L: low-dose group, M: medium-dose group, H: high-dose group, BUN: blood urea nitrogen, CRE: creatinine, UA: uric acid, FFA: free fatty acids, E0: before exhaustive exercise, E1: immediately after exhaustive exercise, E2: 4-h after exhaustive exercise.

Serum levels of protein metabolic markers such as BUN (Figure 1A), CRE (Figure 1B), and UA (Figure 1C) were increased in all the groups immediately after exhaustive exercise (E1) compared to those before exhaustive exercise (E0), while serum CRE and UA levels were decreased 4-h after exhaustive exercise (E2) compared to those at E1. Serum BUN level was still maintained higher at E2 compared to those at E0. However, serum BUN, CRE, and UA levels were not different at any time points among the four groups. Serum ammonia (Figure 1D) and FFA levels (Figure 1E) were also not different at time point E2.

### CHP supplement decreased muscle glycogen after exhaustive exercise

**Figure 2:**
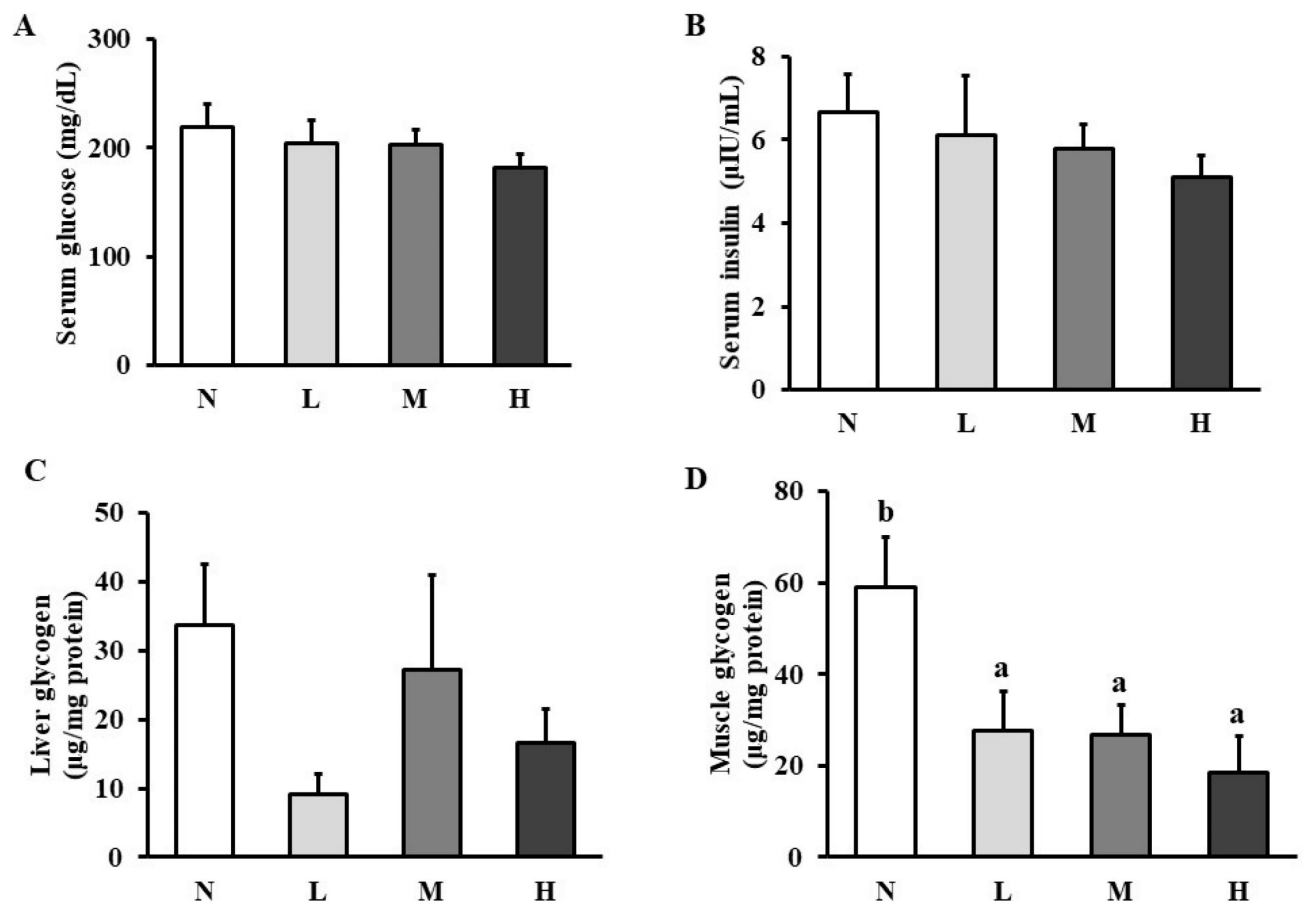
Effect of CHP on carbohydrate metabolism. (A) serum glucose, (B) serum insulin, (C) liver glycogen, and (D) muscle glycogen in exhaustive exercise rats. Data are indicated as mean ± SEM (*n* = 8). Values in different groups with different superscript letters (a, b) were significantly different at *p* < 0.05 by one-way ANOVA and Duncan's new multiple range test. CHP: Chinese herb powder, N: normal group, L: low-dose group, M: medium-dose group, H: high-dose group.

No significant differences were shown in serum glucose (Figure 2A), insulin (Figure 2B), and liver glycogen (Figure 2C) among the four groups. However, muscle glycogen level was significantly reduced in all the intervention groups compared to that in the normal group (Figure 2D).

### CHP supplement did not recover tissue damage after exhaustive exercise

**Figure 3:**
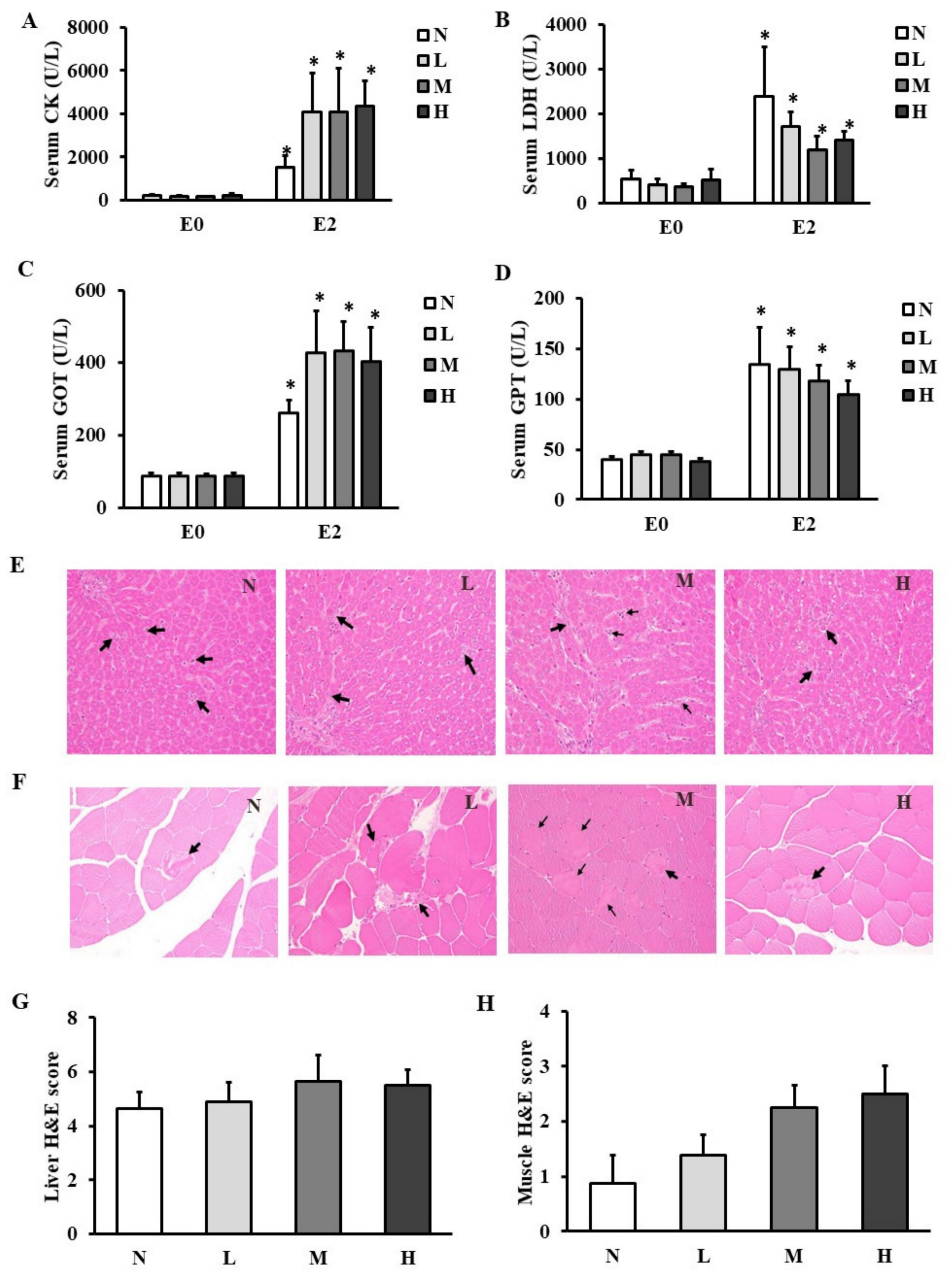
Effect of CHP on tissue damage markers and histopathology. (A) serum CK activity, (B) serum LDH activity, (C) serum GOT activity, and (D) serum GPT activity, (E) liver histopathological staining, (F) muscle histopathological staining, (G) liver total score, and (H) muscle total score in exhaustive rats. Data are indicated as mean ± SEM (*n* = 8). Black arrows represent apoptosis of the hepatocytes and degenerated myofibers in liver and muscles, respectively. All the values were not statistically different among the four groups. ^*^p < 0.05 vs E0. CHP: Chinese herb powder, N: normal group, L: low-dose group, M: medium-dose group, H: high-dose group, CK: creatine kinase, LDH: lactate dehydrogenase, GOT: glutamic oxaloacetic transaminase, GPT: glutamic pyruvic transaminase, E0: before exhaustive exercise, E1: immediately after exhaustive exercise, E2: 4-h after exhaustive exercise, H&E: hematoxylin and eosin.

The activities of tissue damage biochemical markers in muscles such as CK (Figure 3A) and LDH (Figure 3B) and in liver such as GOT (Figure 3C) and GPT (Figure 3D) were elevated at E2 compared to those at E0 in all the groups. However, serum CK, LDH, GOT, and GPT activities were not different among the four groups at any time points. The histopathological staining showed apoptosis in liver (Figure 3E) and degenerated myofibers in gastrocnemius muscles (Figure 3F), but the total pathological scores in liver (Figure 3G) and gastrocnemius muscles (Figure 3H) were not changed among the four groups.

### CHP supplement increased serum SOD activity

Lipid peroxides MDA levels in serum, liver, and gastrocnemius muscles were not altered among the four groups (Table 3). Serum SOD activity tended to be increased after 30-day CHP supplement (*p* = 0.0573). The high-dose CHP group significantly increased serum SOD activity compared to the control group (*p* < 0.05). However, SOD activity in liver and gastrocnemius muscles was not different among the four groups.

### CHP supplement decreased liver IL-6

Liver IL-6 level tended to be reduced in the intervention groups (*p* = 0.0576), but not significantly changed in gastrocnemius muscles among the four groups (Table 3). The medium- and high-dose CHP groups significantly decreased liver IL-6 levels compared to the control group (*p* < 0.05).

## Discussion

The dosage of the active components in the high-dose group of our study was approximately 15% 15, 12% 29, 30, and 2% 31 of the effective dosage for ginsenosides, salidroside, and syringin used in the previous studies, respectively. The dosage of CHP in our study was used because the recommended dosage of CHP for humans by Infinitus (China) Co. Ltd. (Guangzhou, China) was set at 3 g/day, and the dose was equivalent to 50 mg/kg for a 60-kg adult. The dose for rats was 310 mg/kg converted by multiplying by 6.2, a conversion ratio between rats and humans. According to the recommended dosage for evaluating the effects of health food by Taiwan Food and Drug Administration, we designed additional two doses as two and five times of the recommended dosage corresponding to the medium and high dosage, respectively. No dose-related adverse effects of CHP were observed in tissue damage. Therefore, this dosage was considered to be safe to observe whether the anti-fatigue effect could be achieved. We found that the high dose of CHP increased the running time to exhaustion indicating that high-dose CHP could have a synergistic effect.

Exercise endurance is a direct and objective variable to observe fatigue at the macroscopic level, therefore, the time to exhaustion for running treadmill modified from a previous study 32 was used to assess exercise endurance in this study.

Short-term exercise could increase lactate formation by anaerobic glycolysis, and lactate accumulation could decrease blood pH value and further inhibit muscle contraction ability 33. Therefore, enhancing lactate clearance could help fatigue recovery and improve exercise performance 34. Previous studies observed that mice given ginsenoside Rb1 (50-100 mg/kg bw) or salidroside (180-720 mg/kg bw) intragastrically for 28 days or 15 days increased lactate clearance compared to the control group after forced swimming test 16, 19. In our study, CHP intervention lowered serum lactate level in exhaustive exercise rats, suggesting that CHP was capable of promoting lactate clearance. Two potential mechanisms including lactate transport by monocarboxylate transporter (MCT) 35 and lactate utilization via gluconeogenesis by Cori's cycle 36 could be possibly correlated to lactate clearance. We speculated that CHP might increase the expression of monocarboxylate transporter MCT1 and/or MCT4, and/or upregulate lactate uptake into the liver for utilization.

Blood glucose level as an important parameter reflected exercise performance and fatigue responses 22. When the duration of endurance exercise was increased, blood glucose could be maintained by the breakdown of liver glycogen, and metabolized as an energy source in muscles, therefore, low blood glucose level could affect exercise performance 37, 38. Blood glucose level was gradually reduced from the peak at 40 min after treadmill runining exercise and continuously decreased toward exhausion between 40 min and 160 min after exercise, and glycogen levels in liver and muscles were low in exhaustive rats at 120 min after exercise 39. Therefore, preventing a decrease in blood glucose and/or increasing the content of liver and muscle glycogen storage during exercise are crucial factors to enhance exercise performance. Though no significant changes in hepatic glycogen were found in CHP administered rats, CHP intervention decreased glycogen level in gastrocnemius muscles. The high-dose CHP group had the longest time to exhaustion and lower muscle glycogen content, indicating greater consumption of muscle glycogen for prolonging exercise duration to maintain glucose level. The potential mechanism for the decrease in muscle glycogen content after CHP supplement is still not clear, but we infer it from the results of glucose levels in the blood. Serum glucose levels were not significantly different among the four groups at four hours after exhaustive running test, suggesting that serum glucose levels could be maintained after CHP supplement via the utilization of muscle glycogen. However, in order to figure out the fluctuation of serum glucose during exercise, a further study is needed to monitor the changes of blood glucose and muscle glycogen levels for understanding glycogen utilization in muscles at different time points during exercise 40, 41.

Intense or prolonged exercise could cause oxidative damage in muscles, and lipid peroxidation product MDA level could be correspondingly increased 42, which was commonly used as an indicator of lipid peroxidation 16. However, our study revealed that CHP intervention did not alter MDA levels in rat serum, liver, and gastrocnemius muscles. Increased MDA levels in tissues after exhaustive exercise still remained controversial because hepatic MDA level was not altered after acute exhaustive exercise compared to that after chronic exercise 43. Our study also found that CHP intervention tended to increase serum SOD activity. Oral administration of salidroside (15-30 mg/kg bw) for 14 days prior to exhaustive swimming exercise increased nuclear factor erythroid 2-related factor 2 (Nrf2) as a transcription activator of antioxidant enzymes such as SOD in rat myocardial cells, which could lead to reduce oxidative stress and prevent myocardial damage. Additionally, Nrf2 protein expression in rat myocardial cells was positively associated with salidroside intervention concentration 44. Thus, it is reasonable to assume that enhancing Nrf2 protein expression could be correlated to alleviate exercise-induced oxidative stress and further strengthen exercise performance, suggesting that CHP intervention containing salidroside might regulate antioxidative pathway to achieve anti-fatigue effect. The study for the correlation of CHP intervention and Nrf2 related protein expression is required to validate the possible mechanism. However, our study did not find the changes of SOD activity in liver and skeletal muscles among the four groups, even SOD activity in skeletal muscles tended to be increased after high-dose CHP intervention but still not significantly different. A pervious study revealed that SOD activity between serum and liver was weakly correlated (*r* = 0.21, *p* < 0.05), but not associated between serum and skeletal muscles (*p* > 0.05) in physiologically normal rats 45, indicating that SOD activity could not be consistent between serum and tissues. Moreover, we cannot rule out the possible alteration in SOD activity in the certain isoenzyme in tissues after CHP intervention because we only measured the activity of total SOD. There are three SOD isoenzymes in mammals, Cu-Zn SOD (SOD1) in cytosol, Mn-SOD (SOD2) in mitochondrial matrix, and extracellular Cu-Zn SOD (SOD3) secreted from the tissue to the extracellular space 46. Additionally, we speculate that SOD activity in serum or tissues could be altered differently before and after exhaustive exercise against oxidative stress, and changed earlier in serum compared to that in liver and skeletal muscles.

Excessive or prolonged exercise training without sufficient recovery time could promote elevated pro-inflammatory cytokines such as IL-1β, IL-6, and tumor necrosis factor-α, and accompany with fatigue and muscle damage 47. The infiltration of inflammatory cells in the liver lobules was observed in exhaustive exercise rats 48. Additionally, a previous study found that rat plasma IL-6 concentration was significantly increased at the end of exhaustive exercise, but the level was dropped at 2 hours after exercise, suggesting that exhaustive exercise could increase plasma pro-inflammatory IL-6 levels 49. Higher doses of CHP intervention decreased liver IL-6 concentration in our study, indicating that CHP could potentially have anti-inflammatory action in liver. However, IL-6 level in rat gastrocnemius muscles was not changed by CHP intervention. Pro-inflammatory IL-6 derived from skeletal muscles also plays a significant role in muscle recovery after exercise via promoting the activation of muscle satellite cells, increasing muscle growth, stimulating angiogenesis, and enhancing the uptake of glucose and lipolysis in skeletal muscles 50. Hypothetically, IL-6 in the gastrocnemius muscles could act differently from that in liver, speculating that skeletal muscles might still be in a repair phase after exhaustive exercise and require IL-6 for muscle regeneration.

## Conclusions

This study shows that fatigue marker serum lactate level, energy metabolic parameters serum levels of BUN, CRE and UA, and tissue damage markers serum activities of CK, LDH, GOT, and GPT are elevated after exercise. Correspondingly, the histopathological lesions are observed in both liver and gastrocnemius muscles in exhaustive exercise rats. Supplementation with high-dose CHP (1550 mg/kg bw) containing ginsenosides, salidroside, and syringin for 30 days in rats prolongs the time to exhaustion, decreases serum lactate level, increases serum SOD activity, and decreases liver IL-6 concentration, suggesting that CHP exerts anti-fatigue action on prolonging the time to exhaustion via the improvement of lactate clearance, and to a lesser extent, the enhancement of antioxidation and anti-inflammation.

## Supplementary Material

Supplementary figure.

## Figures and Tables

**Table 1:** Effect of CHP supplement on body weight and exercise performance

Variable	N	L	M	H
Initial body weight, g	248.1 ± 4.9	247.9 ± 5.3	249.8 ± 1.5	244.9 ± 5.0
Final body weight, g	399.5 ± 8.2	411.0 ± 12.7	384.3 ± 12.4	394.4 ± 7.9
Grip strength at W0, g	884 ± 46	792 ± 46	921 ± 39	916 ± 50
Grip strength at W4, g	1346 ± 46	1229 ± 50	1333 ± 70	1341 ± 60
Δ Grip strength, g	462 ± 63	436 ± 34	411 ± 67	425 ± 54
Grip strength/bw at W0	3.6 ± 0.1	3.2 ± 0.2	3.7 ± 0.2	3.7 ± 0.2
Grip strength/bw at W4	3.5 ± 0.1	3.1 ± 0.1	3.6 ± 0.2	3.5 ± 0.2
Time to exhaustion, min	48.4 ± 1.5^a^	49.2 ± 2.4^a^	47.9 ± 1.1^a^	58.6 ± 5.1^b^

Data are indicated as mean ± SEM (*n* = 8). Values in different groups with different superscript letters (a, b) were significantly different at *p* < 0.05, and those without superscript letters were not statistically different among the four groups. CHP: Chinese herb powder, N: normal group, L: low-dose group, M: medium-dose group, H: high-dose group, W: week, bw: body weight.

**Table 2:** Effect of CHP supplement on serum lactate level

Time Point	N	L	M	H
	Lactate (nmol/μL)			
Before exercise (L0)	3.2 ± 0.4	3.0 ± 0.4	2.7 ± 0.2	2.5 ± 0.3
After exercise (L1)	4.7 ± 0.4^*^	3.7 ± 0.3^*^	3.9 ± 0.4^*^	3.8 ± 0.2^*^
L1/L0	1.6 ± 0.2	1.5 ± 0.3	1.5 ± 0.1	1.6 ± 0.2
After 30-min rest (L2)	3.5 ± 0.3^ #,b^	2.6 ± 0.2^#,a^	2.3 ± 0.2^#,a^	2.8 ± 0.2^#,a^
L2/L0	1.1 ± 0.1	1.0 ± 0.1	0.9 ± 0.1	1.2 ± 0.2

Data are indicated as mean ± SEM (*n* = 8). Values in different groups with different superscript letters (a, b) were significantly different at *p* < 0.05, and those without superscript letters were not statistically different among the four groups.^ *^p < 0.05 vs L0 and ^#^*p* < 0.05 vs L1. CHP: Chinese herb powder, N: normal group, L: low-dose group, M: medium-dose group, H: high-dose group.

**Table 3:** Effect of CHP supplement on oxidative stress or inflammation markers

Variable	N	L	M	H
Oxidative stress				
Serum MDA, μM	15.0 ± 2.4	13.6 ± 2.4	11.5 ± 1.7	10.4 ± 1.1
Liver MDA, ng/mg	71.2 ± 7.1	61.1 ± 4.5	65.3 ± 7.5	70.1 ± 5.5
Muscle MDA, ng/mg	78.0 ± 7.5	86.2 ± 7.1	85.4 ± 10.6	95.1 ± 9.8
Serum SOD, U/mL	4.6 ± 1.5^a^	11.0 ± 2.3^ab^	11.3 ± 2.1^ab^	15.1 ± 3.8^b^
Liver SOD, ng/mg	34.6 ± 5.0	32.4 ± 11.3	27.4 ± 7.7	34.4 ± 6.7
Muscle SOD, ng/mg	12.1 ± 3.4	12.0 ± 4.7	10.6 ± 4.2	19.0 ± 5.5
Inflammation				
Liver IL-6, ng/mg protein	29.2 ± 5.3^a^	23.8 ± 2.9^ab^	17.5 ± 1.7^b^	18.8 ± 1.1^b^
Muscle IL-6, ng/mg protein	0.9 ± 0.1	1.1 ± 0.1	1.1 ± 0.1	0.8 ± 0.1

Data are indicated as mean ± SEM (*n* = 8). Values not sharing the same superscript indicate statistical significance (*p* < 0.05), and those in the same row without superscript letters were not statistically different among the four groups. CHP: Chinese herb powder, N: normal group, L: low-dose group, M: medium-dose group, H: high-dose group, MDA: malondialdehyde, SOD: superoxide dismutase, IL-6: interleukin 6.

## Abbreviations

BUN: blood urea nitrogen
CHP: Chinese herb powder
CK: creatine kinase
CRE: creatinine
ELISA: enzyme-linked immunosorbent assay
FFA: free fatty acids
GOT: glutamic oxaloacetic transaminase
GPT: glutamic pyruvic transaminase
IL-6: interleukin-6
LDH: lactate dehydrogenase
MDA: malondialdehyde
Nrf2: erythroid 2-related factor 2
SOD: superoxide dismutase
UA: uric acid
