# Supplementary material for: Ginsenosides, salidroside, and syringin complex exhibits anti-fatigue in exhaustive exercise rats
Source: Int J Med Sci. 2025 Jan 1;22(1):17–26. doi: 10.7150/ijms.99889 (PMC11659827; doi:10.7150/ijms.99889)
Supplement: Supplementary file 1 — Supplementary figure. [file ijmsv22p0017s1.pdf]

Supplementary Figure

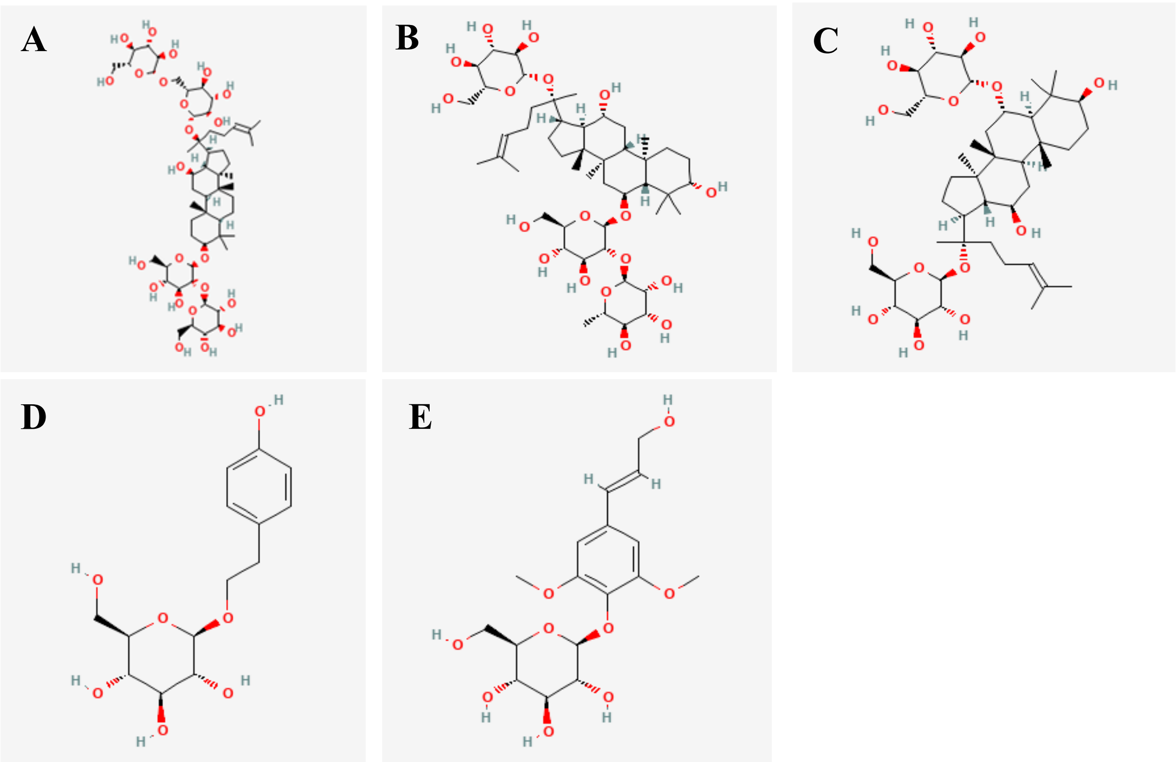

Figure S1. The chemical structures of the active components in Chinese herb powder. (A) ginsenoside Rb1 (IUPAC: (2R,3R,4S,5S,6R)-2-[[(2R,3S,4S,5R,6S)-6-[(2S)-2-[(3S,5R,8R,9R,10R,12R,13R,14R,17S)-3-[(2R,3R,4S,5S,6R)-4,5-dihydroxy-6-(hydroxymethyl)-3-[(2S,3R,4S,5S,6R)-3,4,5-trihydroxy-6-(hydroxymethyl)oxan-2-yl]oxyoxan-2-yl]oxy-12-hydroxy-4,4,8,10,14-pentamethyl-2,3,5,6,7,9,11,12,13,15,16,17-dodecahydro-1H-cyclopenta[a]phenanthren-17-yl]-6-methylhept-5-en-2-yl]oxy-3,4,5-trihydroxyoxan-2-yl]methoxy]-6-(hydroxymethyl)oxane-3,4,5-triol), (B) ginsenoside Re (IUPAC: (2S,3R,4R,5R,6S)-2-[(2R,3R,4S,5S,6R)-2-[[(3S,5R,6S,8R,9R,10R,12R,13R,14R,17S)-3,12-dihydroxy-4,4,8,10,14-pentamethyl-17-[(2S)-6-methyl-2-[(2S,3R,4S,5S,6R)-3,4,5-trihydroxy-6-(hydroxymethyl)oxan-2-yl]oxyhept-

5-en-2-yl]-2,3,5,6,7,9,11,12,13,15,16,17-dodecahydro-1H-cyclopenta[a]phenanthren-6-yl]oxy]-4,5-dihydroxy-6-(hydroxymethyl)oxan-3-yl]oxy-6-methyloxane-3,4,5-triol), (C) ginsenoside Rg1 (IUPAC: (2R,3R,4S,5S,6R)-2-[[(3S,5R,6S,8R,9R,10R,12R,13R,14R,17S)-3,12-dihydroxy-4,4,8,10,14-pentamethyl-17-[(2S)-6-methyl-2-[(2S,3R,4S,5S,6R)-3,4,5-trihydroxy-6-(hydroxymethyl)oxan-2-yl]oxyhept-5-en-2-yl]-2,3,5,6,7,9,11,12,13,15,16,17-dodecahydro-1H-cyclopenta[a]phenanthren-6-yl]oxy]-6-(hydroxymethyl)oxane-3,4,5-triol), (D) salidroside (IUPAC: (2R,3S,4S,5R,6R)-2-(hydroxymethyl)-6-[2-(4-hydroxyphenyl) ethoxy] oxane-3,4,5-triol), and (E) syringin (IUPAC: (2R,3S,4S,5R,6S)-2-(hydroxymethyl)-6-[4-[(E)-3-hydroxyprop-1-enyl]-2,6-dimethoxyphenoxy]oxane-3,4,5-triol).
